# Light Spectra, a Promising Tool to Modulate *Ulva lacinulata* Productivity and Composition

**DOI:** 10.3390/md22090404

**Published:** 2024-09-03

**Authors:** Helena M. Amaro, Fernando Pagels, Rosa Melo, Antoine Fort, Ronan Sulpice, Graciliana Lopes, Isabel Costa, Isabel Sousa-Pinto

**Affiliations:** 1 Interdisciplinary Centre of Marine and Environmental Research (CIIMAR/CIMAR-LA), University of Porto, Terminal de Cruzeiros de Leixões, Avenida General Norton de Matos s/n, 4450-208 Matosinhos, Portugal; lena.amaro@gmail.com (H.M.A.); fernandopagels@gmail.com (F.P.); rmelo@ciimar.up.pt (R.M.); glopes@ciimar.up.pt (G.L.); ispinto@ciimar.up.pt (I.S.-P.); 2 FCUP—Faculty of Sciences, University of Porto, Rua do Campo Alegre s/n, 4169-007 Porto, Portugal; 3 Department of Bioveterinary and Microbial Sciences, Technological University of the Shannon, Midlands, N37 HD68 Athlone, Ireland; antoine.fort@tus.ie; 4 Plant Systems Biology Lab, School of Biological & Chemical Sciences, MaREI Centre for Marine, Climate and Energy, Ryan Institute, University of Galway, H91 TK33 Galway, Ireland; ronan.sulpice@nuigalway.ie; 5 ICBAS—Instituto de Ciências Biomédicas Abel Salazar, University of Porto, Rua Jorge Viterbo Ferreira 228, 4050-313 Porto, Portugal

**Keywords:** seaweed, carotenoids, antioxidant, bioactive, LED

## Abstract

Light quality is a key factor affecting algal growth and biomass composition, particularly pigments such as carotenoids, known for their antioxidant properties. Light-emitting diodes (LEDs) are becoming a cost-effective solution for indoor seaweed production when compared to fluorescent bulbs, allowing full control of the light spectra. However, knowledge of its effects on *Ulva* biomass production is still scarce. In this study, we investigated the effects of LEDs on the phenotype of an *Ulva lacinulata* strain, collected on the Northern Portuguese coast. Effects of white (W), green (G), red (R), and blue (B) LEDs were evaluated for growth (fresh weight and area), photosynthetic activity, sporulation, and content of pigments and antioxidant compounds. The results showed that there were no significant differences in terms of fresh weight accumulation and reduced sporulation among the tested LEDs, while W light induced the highest expansion rate. Under G, *U. lacinulata* attained a quicker photoacclimation, and the highest content of pigments and total antioxidant activity; but with R and W, antioxidant compounds against the specific radicals O_2_^•−^ and ^•^NO were produced in a higher content when compared to other LEDs. Altogether, this study demonstrated that it is possible to modulate the bioactive properties of *U. lacinulata* by using W, R, and G light, opening the path to the production of biomass tailored for specific nutraceutical applications.

## 1. Introduction

*Ulva* species are among the most cultivated Chlorophyta in the world, mostly due to their global distribution in coastal intertidal habitats, high biomass productivity, environmental tolerance, and ability to produce high amounts of bioactive compounds [1].

Despite the growing demand for macroalgae in the EU markets, macroalgae production in Europe remains very limited in comparison to the world’s largest producers in Asia [2,3]. Traditionally, *Ulva* spp. are harvested in the wild or produced in ponds; however, due to seasonality effects and natural variation, the growth, biomass yield and composition of *Ulva* fluctuate considerably [4,5,6]. All this together is a challenge for macroalgae commercialization. Moreover, the Committee for Standardization (CEN) demands standards and practices for algae and algae-based products, particularly concerning specifications for sampling methods and algal products used in different industry sectors, such as food, feed, and cosmetics. Also, special requirements are set for the labelling and marketing of macroalgae and macroalgae products sold as food, since they are included in Regulation (EU) No 1379/2013 [7]. To overcome this challenge, the control of biotic and abiotic factors, like light, could provide a better control on both biomass productivity and quality [2,8], particularly crucial in the case of *Ulva*, whose market is still underexploited, enhancing the suitability and attractiveness of *Ulva* for the nutraceuticals market [2].

Light intensity and quality are key factors that influence algal growth and biomass composition. Algae are able to adapt to different light intensities inducing a variation in thylakoid composition, particularly antennae size and number of photosystem complexes [9,10]. Light quality plays an important role in regulating development, morphogenesis, growth, and photosynthesis in seaweeds. Photosynthesis provides carbon backbones and energy for growth and cellular metabolism [10], influencing physiological processes, such as sporulation and spore release. In general terms, it was reported that blue light has a stimulating effect on reproduction and red light an inhibitory effect; however, this is very species-dependent [9,11]. For example, in Rhodophyta (red seaweeds), such as *Pyropia yezoensis*, red light can stimulate division and induce reproduction, as observed in *Gracilaria birdiae* [12,13]. On the other hand, in Phaeophyceae (brown seaweeds), such as *Laminaria hyperborea*, red light affects pigment composition, increasing the ratio of fucoxanthin/Chl *a* and Chl *c*/Chl *a* [14]. However, for Chlorophyta, fewer studies have been carried out and some of them present different results, especially regarding responses to blue or red light. In some recent studies, higher growth rates were observed under red than blue light [15], whereas in another, blue light induced the same effect on specific growth rate (SGR) as fluorescent light [16]. In this latter study, blue light also induced an increase in antioxidant capacity.

Light-emitting diodes (LEDs) are becoming an advanced and economical technology in indoor algal aquaculture, suitable for replacement of conventional fluorescent lamps [17,18,19]. This technology has several advantages besides low radiant heat output and long lifetime, namely it offers a wide range of emission colours with a narrow emission spectrum (20–30 nm at half-peak height), allowing precise control of light quality. Additionally, it allows the adjustment of light intensity throughout the day, simulating the natural photoperiod [20]. However, the use of LEDs in seaweed cultivation is still uncommon, particularly for production of green algae biomass with high content of bioactive compounds [16]. Most studies evaluating the effects of LEDs on seaweed cultivation only focus on physiological parameters, for example, growth rate and photosynthetic pigment synthesis [16,21,22,23], without considering an integrated study that also monitors other parameters such as photosynthesis rate and production of bioactive compounds (e.g., antioxidants). Thus, the objective of this study was to study the effects of LEDs on *U. lacinulata* growth and composition, envisioning nutraceutical market applications. An integrated study was performed by monitoring growth (fresh weight and surface area), photosynthetic efficiency, sporulation, pigment profile and content, and production of antioxidant compounds in response to the use of white (W), green (G), red (R), and blue (B) LEDs.

## 2. Results

### 2.1. Effects of Light Quality on U. lacinulata Growth and Composition

**Figure 1:**
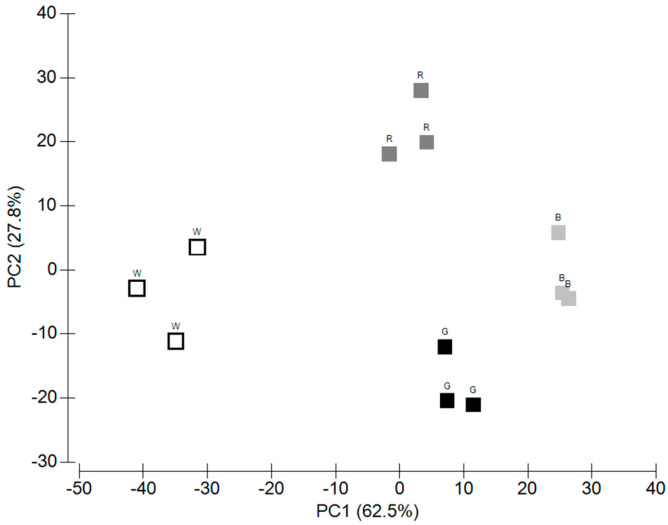
Result of a Principal Component (PC) analysis applied to all parameters monitored in *U. lacinulata* where different responses were observed according to the LED qualities. ■ Blue, ■ Green, ■ Red, and □ White.

To explore the effects of light quality on the different variables related to *U. lacinulata* growth and composition, a principal component analysis, including all parameters, i.e., SGR, growth area, and carotenoid and chlorophyll content, was plotted (Figure 1). The first two principal components explained 90.3% of the variance. The first principal component (PC1) was responsible for 62.5% of the total variation and the second one (PC2) for 27.8%.

The first PCA separated W, R + G, and B and the second R, B + W, and G.

#### 2.1.1. Effects of Light Quality on *U. lacinulata* Growth

**Figure 2:**
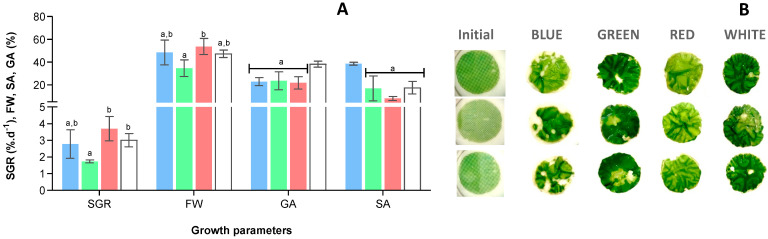
(**A**) Growth rate of *U. lacinulata* after 21 days of cultivation under the different LEDs. ■ Blue, ■ Green, ■ Red, □ White. Bars correspond to the values (average ± standard deviation) obtained for Specific Growth Rate (SGR, % day^−1^), Increase in Fresh Weight (IFW, %), Growth Area (GA, %), and Sporulation Area (SA, %). Bars for the same parameter without a common superscript are significantly different (*p* < 0.05). (**B**) Photographs of the algae at the beginning and at 21 days under each light treatment.

Analysing in detail the values obtained for the different growth parameters (Figure 2), no statistical differences (*p* < 0.05) were detected in specific growth rate between W, R, and B, with values around 3% day^−1^. G was lowest but not statistically different from B. The same observations were made in terms of fresh weight, since *U*. *lacinulata* disks increased, in terms of biomass, by 50% after 21 day of exposure to B, W, and R. In terms of growth area, W seems to have a positive effect, with an increase of 1.7-fold compared to the other conditions tested; but when it comes to SA, a clear statistically significant (*p* < 0.05) negative effect of B was observed, inducing sporulation in 38% of the total disc area. Additionally, a predisposition of R to reduce sporulation is observed, ca. 4.8-fold when compared to B, 8.8 ± 1.7 and 38.5 ± 1.2%, respectively.

#### 2.1.2. Effects of Light Quality on Photosynthetic Activity

The results showed different photosynthetic responses to the different light qualities in *U. lacinulata* (Table 1). In terms of F_v_/F_m_ (photosynthetic efficiency), the cultivation time negatively impacted all cultures, regardless of the treatment. Under W and B, photosynthetic activity reached its lowest value of 0.702 ± 0.003 and 0.696 ± 0.005, respectively, with a decrease starting from day 7 and stabilization from day 14 to day 21. Under R, a less accentuated decrease in photosynthetic yield was also observed, showing higher acclimation in the initial days, with no significant differences between days 7, 14, and 21. Finally, under G, the F_v_/F_m_ value increased from day 0 to day 7 and then decreased until day 21, reaching the same value found in R cultures (0.730 ± 0.005).

In terms of the electron transport rate (Table 1), all treatments induced a decrease on the maximum ETR, and presented no statistical differences between treatments on day 21 (74.2 ± 4.65 μmol_electrons_ m^−2^ s^−1^). The decrease in ETR_max_ was faster under W, G, and B, and it took longer to decrease under R. In terms of saturation light intensity (Table 1), all treatments induced a decrease on E_k_ (1.5 to 1.7-fold), G and R being the treatments with higher reduction in E_k_ (1.7-fold). Changes on E_k_ are usually related to the exposure and sensibility of the algae to the light conditions.

Regarding NPQ_max_ (Table 1), no changes with time were observed under W with an average of 0.687 ± 0.042, while a fluctuation on the values over time was observed in R, G, and B. The algae grown under G seem to be the most affected as decreases of NPQ_max_ in 1.5-fold were observed; while under R the NPQ_max_ increased 1.3-fold on day 7 and 14, but decreased to the initial value on day 21. Finally, under B, the *U*. *lacinulata* showed the lowest value of all conditions at day 14 (0.343 ± 0.047), although on the other days it remained constant at the initial value.

### 2.2. Effect on Pigment Composition over Time

**Figure 3:**
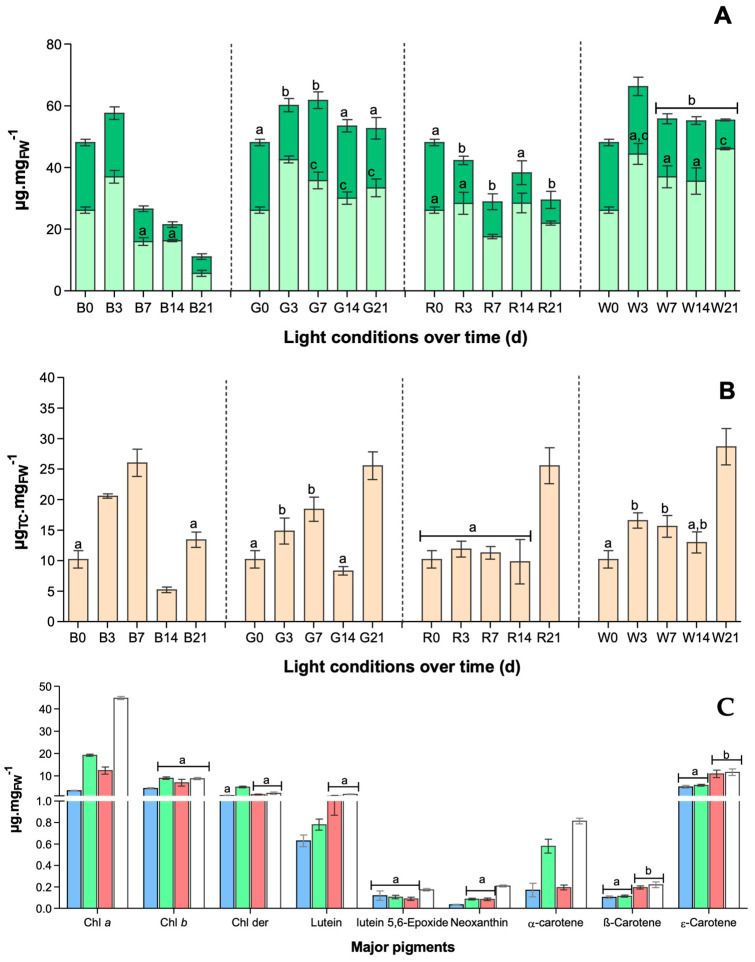
*U. lacinulata* pigment composition over time in terms of (**A**) Total chlorophylls (mean ± stdv) over time, represented by ■ chlorophyll *a*, and ■ chlorophyll *b*, and (**B**) Total carotenoids (mean ± stdv), at each light condition; and after 21 days of exposure. (**C**) Profile and content of main pigments (mean ± stdv) at T21 under ■ Blue, ■ Green, ■ Red, □ White LEDs. Bars with a common superscript for the same parameter are not significantly different (*p* < 0.05).

The effects of each light condition on pigment content were monitored over time in terms of total chlorophyll (Figure 3A) and total carotenoid (Figure 3B) content.

**Figure 4:**
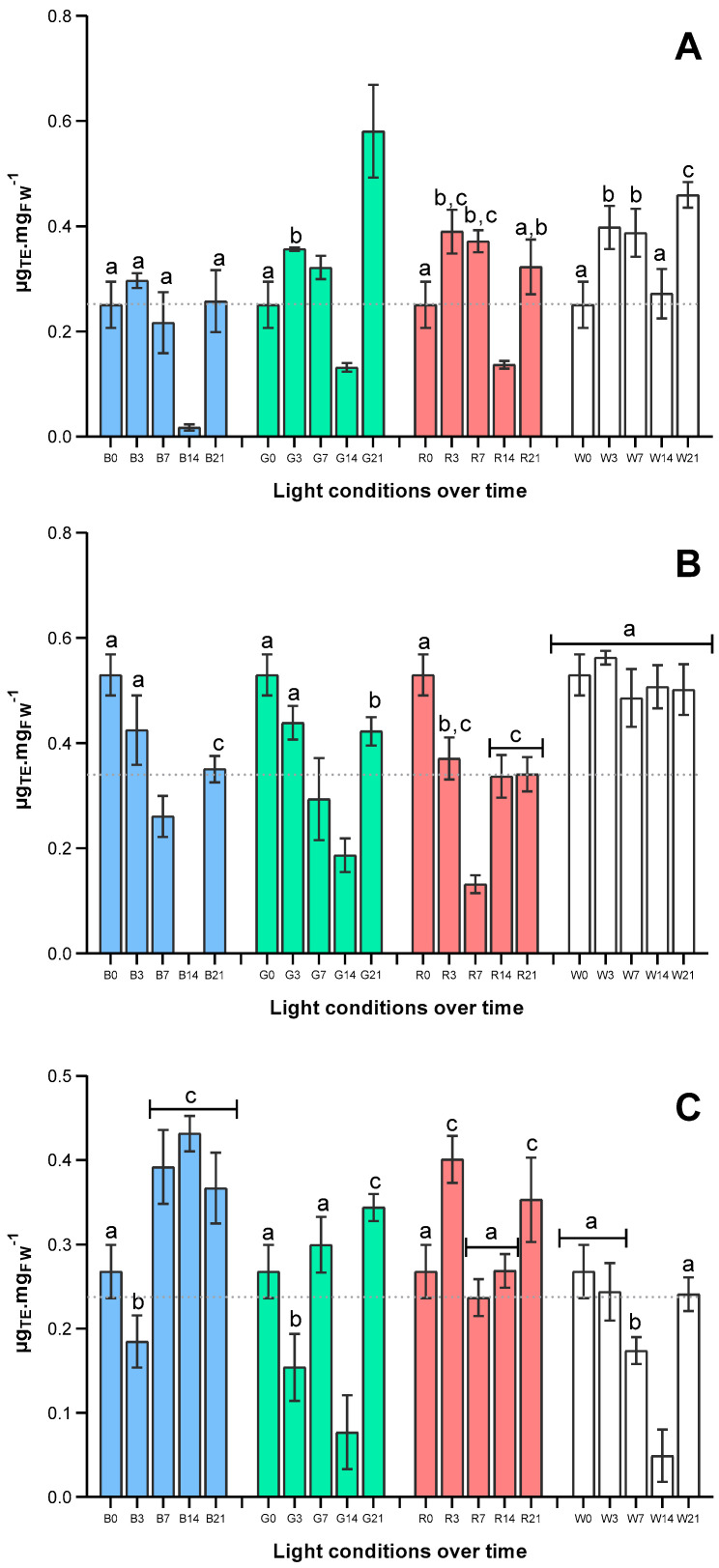
Effects of light quality on antioxidant capacity (µg_TE_ mg_FW_^−1^, mean ± stdv). (**A**) Total antioxidant capacity by ABTS^•+^ assay, (**B**) Specific antioxidant capacity against ^•^NO, and (**C**) Specific antioxidant capacity against O_2_^•−^, obtained at each light condition tested: ■ Blue, ■ Green, ■ Red, □ White. Bars for the same day, without a common superscript, are significantly different (*p* < 0.05).

Generally, chlorophyll content is double total carotenoids but different profiles were observed over 21 days of exposure to the tested light conditions. In terms of chlorophyll (Figure 3A), after 3 days of exposure, its content increased under all light conditions, but a gradual decrease in its content was observed under B and R (4-fold less than at T0 under B). On the contrary, under G and W, the chlorophyll content increased 1.2- and 1.7-fold, respectively, in terms of Chl *a*. Another particular observation was registered, in terms of accessory chlorophylls: under G, the content did not decrease over time, accounting for ca. 40% of total chlorophylls, while in other light conditions it decreased by at least 2-fold. Only one exception occurred with B, in which total chlorophyll content was lower than at T0, while carotenoids were maintained. As expected, over time, an indirect proportionality between chlorophyll and carotenoid content can be perceived, clearly observed under B (Figure 4A). Indeed, in terms of total carotenoids, in all light conditions an increase was observed in the first week (T7) except under R, when the content remained constant. Then, after a decrease in total carotenoid content in the second week (T14), after 21 days of exposure no differences were found in content among G, R, or W, ca. 30 µg_TC_ mg_FW_^−1^.

Observing the pigments profile (Figure 3C), W induces in *U. lacinulata* a higher production of either chlorophyll *a*, lutein-5,6-epoxide, neoxanthin, and α-carotene; and together with R (no statistical differences) higher production of lutein, β-carotene, and ε-carotene than G or B LEDs. On the other hand, B seems to be the light treatment resulting in the lowest contents of all pigments. Overall, carotenoid production is induced by R and W, attaining contents of ca. 2-fold higher compared to B and G. Only one exception is observed, in α-carotene, which together with G seems to increase its production by 5-fold compared to other light qualities.

### 2.3. Effects of Light Quality on Antioxidant Capacity of U. lacinulata

**Figure 5:**
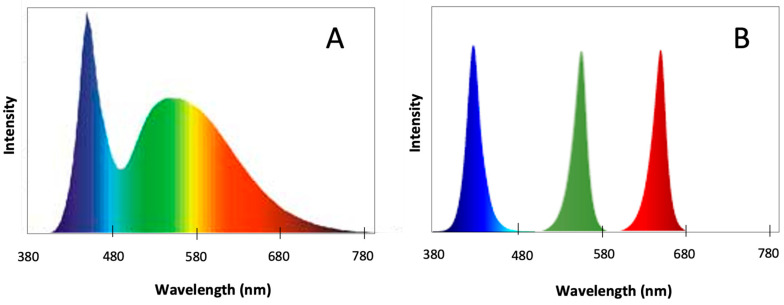
Spectra emission of (**A**) cold white LED and (**B**) monochromatic blue, green, red LEDSs.

The results of total antioxidant capacity of *U. lacinulata* methanolic extracts (via ABTS^•+^ and specific capacity against the biological radicals O_2_^•−^ and ^•^NO) are depicted in Figure 5.

Observing values obtained in terms of total antioxidant capacity of *U. lacinulata* over time (Figure 4A), an increase over time occurred under all light qualities tested except under B. After a global decrease after 2 weeks, at the end of the experiment, *U. lacinulata* exposed to G attained the highest total antioxidant capacity, 0.581 ± 0.058 µgTE mgFW^−1^, ca. 2-fold higher than under B or R, and ca. 1.5-fold than under W.

By contrast, the production of compounds able to scavenge the radical ^•^NO decreased over time under all light conditions tested (Figure 4B), with the exception of W, under which antioxidant capacity remained constant over time. Indeed, at the end of the experiment (T21), W together with G (with no significant differences detected) are the light conditions that attain higher antioxidant capacity, ca. 0.46 µgTE mgFW^−1^, when compared to B or R. However, when it comes to specific antioxidant capacity against O_2_^•−^, depicted in Figure 4C, the results underwent a different variation; *U. lacinulata* under B and R attained higher antioxidant capacity against this radical. Indeed, after 21 days of exposure the antioxidant capacity was 1.5-fold higher than under W, ca. 0.35 µgTE mgFW^−1^, with no statistical differences among B, G, and R.

### 2.4. Pearson Correlation of U. lacinulata Performance Parameters under Each Light Quality

To understand the *U. lacinulata* performance, in terms of how growth, pigment composition, and production of bioactive compounds may or may not be affected together, a Pearson correlation was established (Figure S1) for each light quality (B, R, G, and R LEDs).

Overall, it is clear that *U. lacinulata* has different responses to each light spectrum, and very strong (R^2^ ≥ 0.9) and very significant (*p* < 0.001) correlations were found.

Analysing the growth parameters, with B light, there is a very significant (*p* < 0.001) and positive correlation between the growth parameters SGR, IFW, and F_v_/F_m_ and a strong negative correlation between sporulation and area. This means that although *U. lacinulata* grew under B light, the photosynthetic efficiency is not very high and the quality of biomass is affected due to biomass loss caused by the highest degree of sporulation. Additionally, the B spectrum affects the pigment content in a very strong (R^2^ ≥ 0.9) and very significant (*p* < 0.001) way. Total chlorophyll content is negatively correlated with neoxanthin and β-carotene but they are co-produced with lutein and α-carotene. As expected, there are strong correlations between antioxidant capacity and growth but also between sporulation and T_Chlo_/T_Carot_. This means that the biomass produced under B has a good total antioxidant capacity and this may be related to the stress of sporulation. The specific antioxidant capacity against ^•^NO is correlated with F_v_/F_m_ (R^2^ ≥ 0.9, *p* < 0.001), but negatively correlated with sporulation (R^2^ ≥ 0.9, *p* < 0.001). This means that the low photosynthetic efficiency may have a role in the production of specific antioxidant compounds against ^•^NO radical.

In opposition, the specific antioxidant capacity against O_2_^•−^ is positively strong and significantly correlated (R^2^ ≥ 0.9, *p* < 0.01) with neoxanthin, α-, and β-carotene, indicating that these compounds may be responsible for the antioxidant capacity.

The R LEDs induced in *U. lacinulata* a good equilibrium between growth, photosynthetic efficiency, and sporulation (SGR and F_v_/F_m_) meaning that high photosynthetic efficiency may be responsible for the higher growth rate and lower sporulation, resulting in a very effective way to produce *U. lacinulata*. The effect of R on pigment content was very similar to B. The total content of carotenoids is mainly correlated with neoxanthin and lutein (R^2^ ≥ 0.9, *p* < 0.001), meaning that they are co-produced, whereas a negative correlation between T_Chlo_/T_Carot_ and α-carotene (R^2^ ≥ 0.9, *p* < 0.001) occurred. The total antioxidant capacity of *U. lacinulata* produced with R LED is correlated with IFW and total chlorophylls (R^2^ ≥ 0.9, *p* < 0.001), and negatively correlated with T_Chlo_/T_Carot_ (R^2^ ≥ 0.9, *p* < 0.001), meaning that biomass increase is connected with chlorophylls and α-carotene content. The correlations of specific antioxidant capacity against ^•^NO are very similar with ones obtained with B light, particularly in terms of correlation with growth parameters. On the other hand, O_2_^•−^ inhibition was found to be related to total carotenoids, particularly neoxanthin, lutein, and β-carotene content (R^2^ ≥ 0.9, *p* < 0.001), and negatively correlated with Ulva growth area (R^2^ ≥ 0.9, *p* < 0.001). This means that total carotenoids particularly neoxanthin, lutein, and β-carotene content are responsible for antioxidant capacity against O_2_^•−^ radical but these negatively affected growth in terms of area.

Effects of G on *U. lacinulata* showed a positive correlation between SGR and area growth and sporulation, and a negative one between F_v_/F_m_ and IFW (R^2^ ≥ 0.9, *p* < 0.001), indicating that biomass growth particularly occurs in terms of area but with some sporulation, and that the very high photosynthetic efficiency may not be favouring the increase in fresh weight.

In terms of pigments, a correlation was found among total chlorophylls and T_Chlo_/T_Carot_ (R^2^ ≥ 0.9, *p* < 0.001), but opposite correlated effects between neoxanthin and α-and β-carotene. (R^2^ ≥ 0.9, *p* < 0.001). Under G, total antioxidant capacity (ABTS^•+^) was mostly correlated with F_v_/F_m_ (R^2^ ≥ 0.9, *p* < 0.001), indicating that lutein may be responsible for the total antioxidant capacity and thus a high photosynthetic efficiency favours the antioxidant capacity of *Ulva*.

In another way, besides ^•^NO and O_2_^•−^ antioxidant activity being negatively correlated with ABTS^•+^, (R^2^ ≥ 0.9, *p* < 0.001), this activity is correlated with T_Chlo_/T_Carot_ and negatively, thus, with total carotenoids, SGR, area growth, and sporulation area (R^2^ ≥ 0.9, *p* < 0.001). This means specific antioxidant compounds are not produced under G due to the high photosynthetic efficiency, chlorophyll content, and low increase in fresh weight.

Under W LED, *U. lacinulata* exhibited a negative correlation between SGR and IFW (R^2^ ≥ 0.9, *p* < 0.001), but a very positive one between SGR and IFW, and none with sporulation. This means that W promotes fast growth and increase in biomass and does not influence sporulation.

Similarly to B, under W, positive correlations were found between total carotenoids and β-carotene (R^2^ ≥ 0.9, *p* < 0.001), and total chlorophylls were correlated with neoxanthin and lutein (R^2^ ≥ 0.9, *p* < 0.001), but a negative correlation between T_Chlo_/T_Carot_ and β-carotene (R^2^ ≥ 0.9, *p* < 0.01) was obtained.

A different pattern was observed in terms of antioxidant capacity under W, ABTS^•+^ and O_2_^•−^ results being correlated. Total antioxidant activity (ABTS^•+^) is positively and highly correlated (R^2^ ≥ 0.9, *p* < 0.001) with sporulation and F_v_/F_m_ (R^2^ 0.7–0.8, *p* < 0.05), but negatively correlated with T_Chlo_/T_Carot_ and α-carotene content. This means that sporulation may trigger the production of antioxidant compounds and these compounds are related to photosynthetic efficiency. In another way, ^•^NO antioxidant activity is negatively correlated with SGR and IFW (R^2^ ≥ 0.9, *p* < 0.001) but positively correlated with growth area and total carotenoid content (R^2^ 0.8–0.9, *p* < 0.01), particularly neoxanthin and lutein (R^2^ 0.7–0.8, *p* < 0.05) and β-carotene (R^2^ 0.8–0.9, *p* < 0.01), meaning that these carotenoids may be responsible for such activity. The antioxidant activity against O_2_^•−^ was found to be significantly correlated with sporulation and total antioxidant activity (R^2^ ≥ 0.9, *p* < 0.001). On the other hand, it was negatively correlated (R^2^ 0.8–0.9, *p* < 0.05) with T_Chlo_/T_Carot_ and α-carotene content, meaning that lutein may also be responsible for such activity.

## 3. Discussion

### 3.1. Effects of Light Quality on U. lacinulata Growth

So far, the effects of light quality in green algae are still contradictory, suggesting that it is species-dependent. While some studies demonstrated that blue light in *Ulva pertusa* is more efficient than the red light in developing the thylakoid architecture, keeping the cell maintenance process, morphology [9], and specific growth rate [16], recent results with *Codium tomentosum* revealed that red light is very efficient in biomass and pigment production, similarly to our results [15]. In this study, considering the effects of all growth parameters together, W and R were the light qualities that promoted a better growth of *U. lacinulata*, with no significant differences. This can be explained to some extent by the higher photochemical electron transport rate and thereby a higher efficiency of photochemical action and photosynthetic efficiency observed under R [17].

Surprisingly, B LEDs seemed to induce high rates of sporulation, which is usually observed in brown algae, as reviewed by [24]. However, evidence observed in another species, *Bryopsis plumose*, indicate that gamete discharge was spectrum light-induced, revealing the involvement of a blue light/UV-A-absorbing photoreceptor [25]. Indeed, our results show that the light quality at which less sporulation induction was observed, R, was also the one with the highest average ETR. Although many reports describe that red light induces sporulation in green algae, this was not observed in this study, although it is also observed in other organisms such as *Phytophthora capsici* [26].

### 3.2. Effects of Light Quality on Photosynthetic Activity and Events

Results with *U. lacinulata* show that photosynthetic parameters were affected not only by light quality but also by the culture time. R and G induced a higher photosynthetic activity than other treatments over time. B and W negatively affected it within a week of cultivation. In fact, in long-term cultivation, electron transport rate and light saturation intensity were the same regardless of the light quality, indicating that for a culture with better photosynthetic activity, G and R are the conditions to be applied. Photosynthetic organisms regulate their photo-dependent metabolism and efficiency according to photoreceptor signalling [27,28]. In the present study, *U. lacinulata* showed a better efficiency under G and R. As more energetic lights, they can provide more energy to photosystem excitation, even to the extent that ca. 80% of green light passes through the chloroplast. In fact, green light has been described as an enhancer of the photosynthetic activity in vascular plants, once it is able to pass through the top layer of cells, giving the bottom layer more energy for the photosynthetic metabolism [29]. Indeed, that resulted in the lowest NPQ_max_ 0.415 ± 0.027. That light dilution through the cells allows the organisms to be photosynthetically more efficient than the ones cultivated with blue light, which is only absorbed by the top cells.

Another point to be considered is the time of response for light-dependent reactions. In terms of adaptation time, some responses can depend on how long the organism is exposed to a certain kind of light, with low-fluence responses, as *U*. *lacinulata* grown under R, adapted from day 7, or with high-irradiance responses, taking more time (or in the case, weeks) to reach acclimation [30].

G light did not promote the best SGR performance in this study and no statistical differences were observed in terms of area growth and sporulation, as observed by Gong et al. [17] with *Ulva lactuca*, where green light conditions were more conducive to photosynthesis than blue light.

### 3.3. Pigment Content and Antioxidant Capacity

To understand the responses, in terms of pigment production, of *U. lacinulata* to different light conditions, is important to identify what is known about the light receptors. Senger [31] proposed the existence of a blue light photoreceptor responsible for controlling physiological photoresponses in green algae, a fact later confirmed by Felix Figueroa in *Ulva rigida* [32]. However, it was observed that chlorophyll synthesis by *U. rigida* was also induced by red light pulses and its effect was reversed by far-red light pulses. Knowing that phytochrome was the only pigment to be described as a red-light-absorbing sensor pigment that mediated red and far-red reversible responses, it is possible that a phytochrome or a phytochrome-like photoreceptor could be involved in the induction of chlorophyll synthesis [32].

Actually, the presence of two photoreceptors that allow different responses to red and blue light in *U. rigida* can be supported in terms of adaptation to natural light quality shifting in its natural habitat, the intertidal ecosystem. In clear waters, the action of a blue light photoreceptor would be predominant, whereas in turbid waters, where *Ulva* preferentially grows, the action of a phytochrome system in addition to blue light photoreceptors is essential, since probably under these conditions the very low photon fluence rate of blue light is insufficient in controlling chlorophyll synthesis [32]. The low Chl *a* content after 21 days of exposure to B observed in our strain at 50 µmol photon m^−2^ s^−1^ cannot be explained by low photon fluence. Since the energy provided by B is higher compared to R, one possible explanation maybe that the algae were photostressed, leading to Chl *a* degradation, and to a decrease in this pigment over time reaching the lowest content of Chl *a*, 14.6-fold less than that obtained under W.

Carotenoids are powerful antioxidants that are induced by high light stress to protect cells against photooxidative processes. Previous studies about the effects of light quality on antioxidant compound production revealed that blue light induced its production in green algae such as *U. pertusa* [16] and *Caulerpa lentillifera* [19]. However, there are no reports of the effects of green light on induction of antioxidant compounds in green algae. Moreover, the potential influence of light on carotenoid synthesis and antioxidant activities in seaweeds is understudied [19].

Nevertheless, it is known that the absorption characteristics of algae depend on several other factors, like thallus morphology, or the thickness or structure of the photosynthetic system [33]. Due to the low thickness of *Ulva*, most likely the green wavelength was sufficiently short to be absorbed and retained among cell layers, explaining the high efficiency in pigments production observed in *U. lacinulata* [17], particularly chlorophyll derivate and α-carotene. Additionally, a study by Kang et al. [19] related the content of carotenes to antioxidant capacity via ABTS assay, and in our study a positive correlation with lutein (R^2^ 0.8–0.9, *p* < 0.05) was found, which may be a reason for the high antioxidant capacity of *U. lacinulata* under G. In other way *U. lacinulata* antioxidant activity against ^•^NO and O_2_^•−^ seems to be also strongly correlated with total chlorophylls content (R^2^ ≥ 0.8, *p* < 0.01)

Under W, *U. lacinulata* also revealed high total antioxidant content in ABTS and particularly against ^•^NO radical, although its value after 21 days of exposure was similar to T0. This activity can be clearly negatively correlated with the content of pigments, particularly with T_Chlo_/T_Carot_ and α-carotene content (R^2^ 0.7–0.8, *p* < 0.05). Indeed, given the emission spectra, with a high blue peak, and consistent amounts of green and red regions in W, it is possible to identify the contribution of red light in the activation of phytochrome, resulting in high concentrations of Chl *a* and carotenoids, regulated by blue and red light [34].

*Ulva* sp. is described to produce several compounds with known antioxidant capacities, such as polysaccharides like ulvan and phenolic compounds [16], that were not fully characterized in this study. Hence, the production of these molecules under B, G, or R light qualities could be responsible for the good results in terms of antioxidant capacity against O_2_^•−^ radicals.

## 4. Materials and Methods

### 4.1. Sampling and Ulva Maintenance

*Ulva* samples were collected on the Belinho-Mar rocky shore (−41.59113° N; −8.80576° W), located on the northwest coast of Portugal (Esposende, Braga). The shore is characterized by a wide rocky platform, either emerged or submerged, according to tidal dynamics, with high exposure to waves. The intertidal habitat of Belinho-Mar is characterized by the dominant presence of *Ulva* spp., especially during spring and summer. Specimens were collected and placed in resealable bags onsite, filled with seawater, and kept in a chilled container. At the laboratory, *Ulva* thalli were cleaned with filtered seawater and epiphytes were removed if present. Specimens were maintained for at least two weeks in artificial seawater (34 g L^−1^; Red Sea Coral Pro) enriched with f/2 medium [35], at 16 ± 1 °C, and using fluorescent Biolux lamps (Osram, Premstaetten, Austria) as the source of light with an intensity of 50 µmol photon m^−2^ s^−1^ and light/dark cycles of 16:8 h.

### 4.2. Species Identification

The three samples used in this study were genetically characterised in previous studies that used genetic barcoding (barcodes *rbc*L, ITS1, and *tuf*A, all three showing 100% identity with samples belonging to *U. lacinulata*) [36] and a CAPS assay [37] that confirmed that the samples were pure *U. lacinulata*.

### 4.3. Light Quality Experiments

To assess the effect of different light qualities on the cultivation of *U. lacinulata*, discs from homogeneous areas of thalli were collected with a cork borer, each disc being of ca. 3.14 cm^2^. The discs were placed in a sterilized glass Petri dish containing 40 mL of sterile f/2 medium, restrained by a stainless-steel circle covered with a light permeable mesh.

The three monochromatic LEDs, blue (B) (peak at 440 nm with a range of 420–470 nm), red (R) (peak at 660 nm with a range of 600–700 nm), and green (G) (peak at 525 nm with a range of 513–543 nm) and a polychromatic cold white (W) LED were tested (Figure 5). The effect of each light quality on *U. lacinulata* was evaluated by measuring growth, photosynthetic activity, pigment production, and antioxidant compounds production. For each light quality tested, 15 pseudoreplicates (3 replicates) of *U. lacinulata* were acclimated for two days under Biolux fluorescent lamps at 50 µmol photon m^−2^ s^−1^, and then exposed to each light quality, B, R, G, and W, at 50 µmol photon m^−2^ s^−1^ with light/dark cycles of 16:8 h, at 16 ± 1 °C for 21 days. To monitor *U. lacinulata* growth over time, 3 replicate discs were sampled at days (t) 0, 3, 7, 14, and 21.

### 4.4. Growth Evaluation

Growth was monitored in all light conditions during the experiments. Using a precision scale, the initial fresh weight (t = 0 days) of each replicate was measured. The same procedure was performed at 7, 14, and 21 days of culture.

The mean specific growth rate (SGR) was assessed according to the formula: SGR (% day^−1^) = ln (FW_t_/FW_0_)/t × 100, where FW_0_ is the initial fresh weight (FW) and FW_t_ is the FW after t days.

The growth area determination was implemented by image analysis, using Image J 1.54g software (http://imagej.org, accessed on 29 August 2024) with photos taken at each sampling. This procedure also allowed estimation of the sporulation area, using colour and contrast editing tools provided by the software.

### 4.5. Photosynthetic Activity Measurement

Changes in photosynthetic activity were evaluated by in vivo chlorophyll *a* fluorescence monitoring with Pulse-Amplitude Modulation (PAM) using a Junior-PAM/W fluorimeter (Walz, Effeltrich, Germany) according to [38,39]. Rapid light curves (RLCs) were performed with *Ulva* circles in contact with the fibber (90-degree angle) fixed in the centre of the disc. The measurement was carried out in triplicate, by incubating the algae in darkness for 15 min, and then a rapid light curve (RLC) was initiated, involving a 30 s exposure to 12 incremental irradiances. The alga was exposed to a basal measurement to estimate the minimum fluorescence (Fo), followed by a saturating pulse of 0.8 s (with irradiance higher than 4000 μmol photons m^−2^ s^−1^) to obtain maximal fluorescence from the PSII (Fm) and the F_v_/F_m_ (RLC). Then, it was exposed to 30 s acclimation to different actinic lights, followed by saturation pulse application, resulting in the parameters Ft (fluorescence of actinic light acclimated samples) and Fm’ (maximal fluorescence in the light) reached by the saturation light pulse. Ft and Fm’ were used to calculate Y(II) = (Fm’ − Ft)/Fm’. RLCs were made by calculating the electron transport rate (ETR) for actinic light irradiances with the following formula: ETR = Y(II) × E × f_AQPSII_, where Y(II) is the effective quantum yield and f_AQPSII_ is the fraction of absorbed quanta to PSII, dimensionless, the value f for Chlorophyta being 0.5 according to [21]. Non-photochemical quenching (NPQ) was calculated after each saturating pulse during the RLCs, and NPQ versus irradiance relationships were determined. These curves were then fitted to a mathematical function [40] to find the maximal non-photochemical quenching (NPQ_max_), maximal ETR (ETR_max_), and the irradiance for saturation of ETR (E_k_), expressed in μmol photons m^−2^ s^−1^.

### 4.6. Pigment Composition and Antioxidant Capacity

One of the goals of this study was to evaluate the effects of light quality on *U. lacinulata* biomass composition, considering the benefits for consumers’ health, due to the production of high-value bioactive compounds, such as pigments and other antioxidant compounds. Thus, to assess the biochemical composition of *U. lacinulata*, a cold extraction with methanol (1:40 *w*/*v*) was performed at 2 °C, overnight, until a total depigmentation of the tissue. Extracts were maintained at −20° C until further analyses of pigment and antioxidant capacity.

#### 4.6.1. Pigment Quantification

Pigment quantification was conducted over the course of the experiment, via spectrophotometry on methanol extracts. So, total carotenoids, total chlorophylls, chlorophyll *a*, and chlorophyll *b* were assessed and calculated in terms of the concentration of pigment per fresh weight of algae (µg mgFW^−1^) using the equations developed by [41].

After 21 days of culture, the profile and quantification of carotenoids was determined by High Performance Liquid Chromatography (HPLC) with photodiode array (PDA) detection (Waters Alliance 2695, Milford, MA, USA), as described by [42]. For this purpose, 2 mL of each extract with 100 µL of β-carotenol internal standard (170 mgL^−1^; Sigma, Lisbon, Portugal) was evaporated and then suspended in 200 μL of acetone/acetonitrile (9:1).

The stationary phase was constituted by a 4 × 250 mm Purospher Star RP-18e (5 μm) column (Merck, Darmstadt, Germany) and mobile phase by ethyl acetate (solvent A), and acetonitrile/water at 9:1 (*v*/*v*) (solvent B). An overall flow rate of 1 mL min^−1^ was set with various volumetric ratios of the solvents A and B over the following elution times: 0–31 min (0–60% A); 31–36 min (60% A); 36–38 min (60–100% A); 38–43 min (100% A); 43–50 min (100–0% A); and 50–55 min (0% A). In order to maintain a constant temperature of 25 °C, a column heater (Waters, Milford, MA, USA) was used during the analyses. Spectral data from all peaks were collected in the range 250 to 750 nm and pigments were identified by comparing the retention times and the UV–visible spectrum with the standards. Authentic standards used for HPLC profiling (Extrasynthese, Sigma) consisted of chlorophyll *a*, chlorophyll *b*, lutein, lutein 5,6 epoxide, neoxanthin, α-carotene, β-carotene, and ε-carotene. The results were expressed in terms of the concentration of pigment per fresh weight of algae.

#### 4.6.2. *U. lacinulata* Antioxidant Capacity Assessment

The antioxidant capacity of *U. lacinulata* extracts was evaluated by three spectrophotometric assays: one that assesses the total antioxidant capacity, 2,2′-azino-bis-3-ethylbenzthiazoline-6-sulphonic acid (ABTS^+•^), and two specifics for two biological radicals, superoxide (O_2_^•−^) and nitric oxide (^•^NO), that may be indicative of *U. lacinulata*’s nutraceutical potential.

Each extract was evaporated and suspended in the same volume of phosphate buffer (100 mM, pH 7.4) with 20% dimethyl sulfoxide (DMSO). In order to quantify the antioxidant capacity, a calibration curve using a known antioxidant, Trolox, was established for each method and thus the antioxidant capacity was expressed as Trolox equivalents (TEs) per fresh weight (FW) of biomass—(µgTE mgFW^−1^).

The total antioxidant capacity of each extract was assessed against the radical ABTS and determined in triplicate in a 96-well plate [43]. Briefly, to 63 µL of extract was added 180 µL of ABTS^•+^, and the absorbance was read at 734 nm, after 6 min.

Superoxide radical (O_2_^•−^) scavenging capacity of extracts was determined spectrophotometrically by inducing superoxide radical reduction by nitroblue tetrazolium, at 562 nm [43]. Regarding nitric oxide (^•^NO) scavenging capacity determination, extracts were incubated with sodium nitroprusside in triplicate, for 60 min at room temperature, under ambient light. Griess reagent was added, and the chromophore reaction was undertaken in the dark for 10 min, and absorbance was read at 562 nm [43].

### 4.7. Statistical Analysis

The experimental data were analysed using GraphPad Prism v. 5.0. A first diagnostic unfolded a non-normal distribution of the data; therefore, a 1-way ANOVA with Tukey’s multiple comparisons test was used to assess variances in the several parameters between the different light treatments tested on *U. lacinulata* growth and composition. Since each data point had been replicated, a representative measure of variability was available in all cases to support the mentioned statistical analyses. The impact of light quality on *U. lacinulata* growth and compositions was also assessed by performing a Principal Component Analysis using Primer v6 software (PRIMER-e, Albany, Auckland New Zealand). Also, Pearson correlation of some determined parameters in terms of growth, pigment composition, and antioxidant capacity (specific growth rate, increased percentage of fresh weight, percentage of sporulation, F_v_/F_m_, total carotenoids, total chlorophylls, T_carot_/T_chl_, neoxanthin content, lutein content, α-carotene content, β-carotene content, total and specific antioxidant capacity) was established using GraphPad Prism v. 5.0.

## 5. Conclusions

*U. lacinulata* thalli were demonstrated to be able to adjust their growth and metabolism to several light spectra. Indeed, this artificial illumination seems to be a useful tool to avoid seasonal variability in seaweed biomass production and meet the requirements of the Committee for Standardization.

Different spectra were shown to induce different responses in *U. lacinulata* in terms of growth, and also composition and content of carotenoids and antioxidant compounds.

Among all the LED spectra tested, this study shows that using W allows for high biomass production of *U. lacinulata* due to the high specific growth rate and fresh weight increase with low sporulation levels. Additionally, this biomass has a high content of carotenoids and is rich in specific antioxidant compounds against ^•^NO radicals.

On the other hand, if the purpose is to produce *U. lacinulata* biomass with high antioxidant capacity to be used for nutraceutical purposes, having high total and specific antioxidant capacity against both ^•^NO and O_2_^•−^ radicals, G seems to be the most suitable, although biomass production may be lower.

Furthermore, the study of the *U. lacinulata* responses over time gives important information for further studies, in which a 2-stage production may be considered—a first stage that uses white light to promote a fast production of biomass, and a second stage in which light can be switched to green to induce the production of carotenoids and antioxidant compounds.

Hence, this study demonstrated that it is possible to modulate the bioactive properties of *U. lacinulata* opening the path to the standardized production of biomass tailored to specific nutraceutical applications.

## Figures and Tables

**Table 1:** Photosynthetic parameters (average ± standard deviation, *n* = 9) for *U. lacinulata* grown under different light qualities (blue, B; green, G; red, R; and white, W) as a function of time: maximal quantum yield (F_v_/F_m_), maximum electron transport rate (ETR_max_, μmol_electrons_ m^−2^ s^−1^), saturation light intensity (E_k_, μmol photons m^−2^ s^−1^), and maximum non-photochemical quenching (NPQ_max_). Different lowercase letters show statistically significant differences (*p* < 0.05).

Light Quality	Time	F_v_/F_m_	ETR_max_	E_k_	NPQ_max_
B	0	0.793 ± 0.009 ^a^	150.6 ± 2.6 ^a^	601.9 ± 18.6 ^a^	0.680 ± 0.038 ^a^
	3	0.704 ± 0.004 ^b^	107.3 ± 1.8 ^b^	448.5 ± 8.8 ^b^	0.675 ± 0.028 ^a^
	7	0.738 ± 0.006 ^c^	116.8 ± 2.4 ^b^	496.8 ± 3.6 ^b^	0.622 ± 0.016 ^a^
	14	0.696 ± 0.005 ^b^	64.8 ± 0.2 ^c^	143.5 ± 0.8 ^c^	0.343 ± 0.047 ^b^
	21	0.671 ± 0.013 ^b^	67.2 ± 9.8 ^c,d^	300.7 ± 11.8 ^d^	0.781 ± 0.022 ^c^
G	0	0.782 ± 0.007 ^a^	164.9 ± 2.9 ^a^	624.3 ± 7.1 ^a^	0.689 ± 0.015 ^a^
	3	0.868 ± 0.008 ^d^	118.4 ± 1.2 ^b^	392.8 ± 3.6 ^e^	0.698 ± 0.029 ^a^
	7	0.808 ± 0.005 ^a^	93.6 ± 1.2 ^e^	245.9 ± 3.3 ^d^	0.518 ± 0.053 ^d^
	14	0.758 ± 0.006 ^c^	75.9 ± 2.2 ^d^	193.9 ± 8.9 ^f^	0.510 ± 0.030 ^d^
	21	0.730 ± 0.005 ^c^	76.1 ± 3.2 ^d^	266.5 ± 8.3 ^d^	0.415 ± 0.027 ^b^
R	0	0.794 ± 0.002 ^a^	156.7 ± 3.2 ^a^	614.5 ± 5.5 ^a^	0.672 ± 0.017 ^a^
	3	0.694 ± 0.005 ^b^	117.8 ± 1.3 ^b^	456.3 ± 5.6 ^b^	0.701 ± 0.036 ^a^
	7	0.744 ± 0.003 ^c^	152.1 ± 2.9 ^a^	495.7 ± 3.8 ^b^	0.916 ± 0.043 ^d^
	14	0.752 ± 0.009 ^c^	126.5 ± 2.4 ^f^	488.5 ± 6.3 ^b^	0.866 ± 0.084 ^c,d^
	21	0.729 ± 0.011 ^c^	76.7 ± 3.3 ^d^	266.7 ± 12.3 ^d^	0.672 ± 0.088 ^a^
W	0	0.793 ± 0.005 ^a^	155.1 ± 1.9 ^a^	611.6 ± 1.1 ^a^	0.699 ± 0.023 ^a^
	3	0.815 ± 0.005 ^a^	111.6 ± 1.1 ^b^	339.6 ± 2.9 ^g^	0.696 ± 0.037 ^a^
	7	0.763 ± 0.004 ^c^	116.2 ± 3.6 ^b^	366.5 ± 2.9 ^g^	0.692 ± 0.081 ^a^
	14	0.702 ± 0.003 ^b^	73.1 ± 0.9 ^d^	206.4 ± 4.1 ^f^	0.571 ± 0.061 ^a,d^
	21	0.703 ± 0.008 ^b^	76.8 ± 4.1 ^d^	280.8 ± 3.7 ^d^	0.664 ± 0.012 ^a^

## Data Availability

The original contributions presented in the study are included in the article/Supplementary Material, further inquiries can be directed to the corresponding author/s.

## Supplementary Materials

The following supporting information can be downloaded at: https://www.mdpi.com/article/10.3390/md22090404/s1, Figure S1: Pearson correlation matrix between growth parameters (SGR, IFW, Area, Spor, Fv/Fm), pigments composition (Tcarot, Tchlo, Tchlo/ Tcarot, Neox, Lutein, α-carot, β-carot) and antioxidant capacity (ABTS^•+^, ^•^NO and O2^•−^, obtained under B, R, G and W LEDS. The colour of the intersections represent the correlation coefficient ( 0.7–0.9;  0.8–0.9;  ≥0.9), if is positive (+) or negative (−), and the thickness edge line indicates the significance levels for each comparison, none- *p* < 0.05,  *p* < 0.01, and  *p* < 0.001.
