# Supplementary material for: Light Spectra, a Promising Tool to Modulate Ulva lacinulata Productivity and Composition
Source: Mar Drugs. 2024 Sep 3;22(9):404. doi: 10.3390/md22090404 (PMC11433255; doi:10.3390/md22090404)
Supplement: Supplementary file 1 [file marinedrugs-22-00404-s001.zip › marinedrugs-3181833-supplementary.pdf]

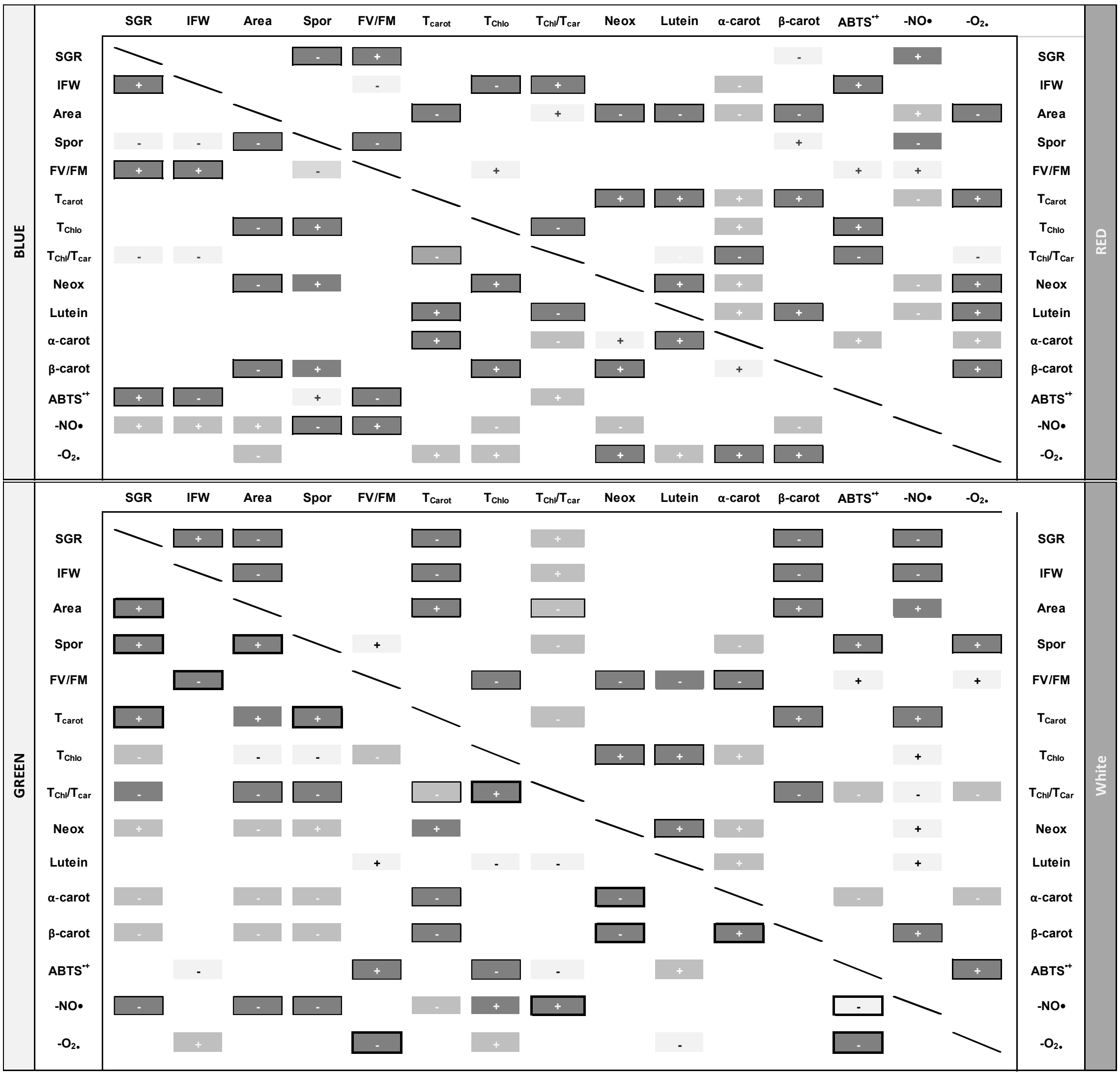

SGR-pecific growth rate; **IFW**- increase percentage of fresh weight; **Area**- percentage of growth area; **Spor**- sporulation area; **Fv/Fm**- maximal quantum yield; **T_{carot}**-total carotenoids; **T_{chlo}**- total chlorophylls; **T_{chlo}/ T_{carot}**- ration between total chlorophylls and carotenoids; **Neox**- neoxanthin content; **Lutein**- lutein content; ***α*-carotene**- *α*-carotene content; **β-carotene**- β-carotene content; **$ABTS^{•+}$**- total antioxidant capacity; •NO - antioxidant capacity against nitric oxide; **$O_2^{•-}$**- antioxidant capacity against superoxide radical.

Figure S1. Pearson correlation matrix between growth parameters (SGR, IFW, Area, Spor, Fv/Fm), pigments composition (T_{carot}, T_{chlo}, T_{chlo}/ T_{carot}, Neox, Lutein, *α*-carot, β-carot) and antioxidant capacity ($ABTS^{•+}$, •NO and $O_2^{•-}$, obtained under B, R, G and W LEDS. The colour of the intersections represent the correlation coefficient (0.7 – 0.9; 0.8–0.9; ≥ 0.9), if is positive (+) or negative (-), and the thickness edge line indicates the significance levels for each comparison, none- *p*< 0.05, *p*<0.01, and *p*<0.001.
